# A pilot study of team-based learning in one-hour pediatrics residency conferences

**DOI:** 10.1186/s12909-019-1702-z

**Published:** 2019-07-18

**Authors:** Anna Volerman, Rachel Stork Poeppelman

**Affiliations:** 1 Departments of Medicine and Pediatrics, University of Chicago, 5841 S. Maryland Ave, MC 2007, Chicago, IL 60637 USA; 2 Department of Pediatric Critical Care, Nationwide Children’s Hospital, 700 Children’s Drive, Columbus, OH 43205 USA

**Keywords:** Team-based learning, Residency, Graduate medical education

## Abstract

**Background:**

Active learning has been shown to improve knowledge retention, facilitate feedback, and motivate learners. Despite this evidence, lecture, a passive mode of instruction, is the most widely utilized instructional method for residency educational conferences. Team-based learning fosters active learning but is infrequently used during residency training.

**Methods:**

Three team-based learning sessions (one introductory and two content-based) were held during noon conferences in a pediatrics residency program. A pre-post static-group design was used to evaluate learner satisfaction and knowledge gains. Additional data was collected about facilitator preparation, session attendance, and readiness assurance test scores. Descriptive statistics and qualitative content analyses were conducted.

**Results:**

Forty-seven residents and students participated (81%, 47 of 58). Prior to the introductory session, the majority of participants (55%) were not familiar with team-based learning. After the three sessions, 65% of residents and students reported high levels of satisfaction with team-based learning. When compared to traditional, lecture-based noon conferences, 76% of participants reported more engagement and 48% perceived more learning in team-based learning sessions. Challenges included low completion rates of the assigned reading prior to the session and abridged discussions due to time limitations during sessions. Each session required 10 hours of preparation for curriculum development.

**Conclusions:**

Team-based learning resulted in greater satisfaction and engagement among learners when compared to lecture-based formats. However, it did not prove to be a feasible instructional method during one-hour residency conferences. Adaptations that promote learner preparation for sessions and overcome time limitations during sessions may improve the feasibility and impact of team-based learning during one-hour conferences.

**Trial registration:**

Not applicable.

**Electronic supplementary material:**

The online version of this article (10.1186/s12909-019-1702-z) contains supplementary material, which is available to authorized users.

## Background

Active learning methods improve knowledge retention, facilitate feedback, and motivate learners [1, 2]. They are also well-suited to foster non-technical skills, such as communication and teamwork. Despite the benefits of active learning, implementation challenges arise in residency due to time constraints. Methods such as flipped classroom and simulation are often time-intensive and difficult to integrate into one-hour conferences [1, 3].

Team-based learning (TBL) is an active learning method that is widely used in medical school education. Each TBL session follows a structured approach: preparation, readiness assurance tests (RATs), and application exercises [4]. Preparation supports foundational knowledge acquisition through readings or videos completed before class. In class, learners’ grasp of the foundational knowledge and preparation is first assessed with an individual readiness assurance test (IRAT). Learners then work through the same test in groups, termed the group readiness assurance test (GRAT), to deepen understanding and make connections through dialogue and debate. Finally, application exercises require learners to work in teams to apply their knowledge to complex real-world problems without one final answer. Discussion is critical to TBL, particularly as a large group following the GRAT and the application exercise, to support peer learning. For each TBL session, teams are kept intact to foster collaboration.

Fewer than 10 studies describe specific TBL curricula in residency programs, including family medicine, internal medicine, pathology, psychiatry, physical medicine and rehabilitation, and surgery; none in pediatrics [5]. Positive outcomes are described in learner satisfaction and engagement for the majority of learners [5, 6]. Due to the emphasis in TBL on covering a smaller breadth of material in greater depth, a subset of resident learners in one study felt TBL was less efficient than lectures [7]. However, studies of TBL in residency do show knowledge gains based on resident self-assessment and significant increases in scores from the IRAT to GRAT [8–10]. The curricula vary in structure and length, with most utilizing 2–3 h blocks for TBL. Only two studies have examined TBL in one-hour conferences. One study applied the TBL structure for a monthly journal club in psychiatry with participants describing high acceptance and perceived benefit for learning clinical appraisal skills. Another study evaluating a year-long general surgery curriculum showed that the TBL format led to improved engagement of learners, greater perception of knowledge gains and the educational experiences, and higher in-training exam scores [9, 11]. Given the positive experiences in other specialties, we aimed to apply TBL in one-hour pediatrics conferences and evaluate feasibility, learner satisfaction, and knowledge acquisition.

## Methods

We implemented TBL in one-hour conferences for a pediatrics residency program at an urban academic medical center. In February 2015, three one-hour TBL sessions were held during the residency noon conferences, replacing the traditionally utilized lectures. Table 1 provides an overview and timeline for the sessions.

**Table 1:** Timeline of team-based learning (TBL) sessions in pediatrics residency noon conferences

TBL component	Content	Planned time
Pre-work	Journal article about topic distributed to residents in person and via email	Outside of class
Introduction	Facilitators introduce session	2 min
Readiness assurance test (RAT)		
IRAT	6–7 multiple choice style questions completed individually	5 min
GRAT	Same questions completed by team	7 min
Discussion	Teams simultaneously share their answers (“report out”), followed by large group discussion of each answer; repeated for each question	10 min
Application exercise		
Explanation	Facilitators explain exercise	1 min
Team	Teams work on the same cases concurrently	8 min
Discussion	Teams simultaneously shared solution, followed by large group discussion for each case; repeated for each case	10 min
Conclusion	Facilitators summarize key points and conclude session	2 min

The three TBL sessions were held within a two-week clinical block to maximize team consistency. Learners were divided into six teams by the facilitators based on the clinical rotation to promote team development. Teams had 4–6 members, ranging from third-year medical students to fourth-year residents; each team had approximately the same number of students, first-year residents, and upper level residents. Faculty members who attended the conferences observed and shared input but did not join teams due to irregular attendance. The two facilitators for all the sessions were a pediatrics faculty member and resident, both with experience in medical education and training in TBL through masters level coursework.

The topics for the TBL sessions were selected based on existing gaps in the residency’s conference curriculum. The first session introduced learners to TBL with a team-building exercise. Two subsequent TBL sessions focused on sports physicals and menstrual disorders. Sessions were developed using the principles of backwards design [12]. Learning objectives and a related application exercise were developed first, with a focus on aligning objectives with the residency’s curriculum and board specifications. The application exercise was developed based on the principles of 4S (significant problem, same problem, specific choice, and simultaneous reporting). Based on the objectives and application exercise, multiple choice questions and pre-reading articles were selected that supported the necessary foundational knowledge. The content of the RAT and application exercises was reviewed by faculty with topic expertise.

Sessions were conducted using the standard TBL structure [4]. Table 2 describes the essential elements of TBL included in each session, based on the guidelines for reporting of TBL [13]. Pre-class preparation consisted of reading a journal article about the specific topic, distributed at the prior session and via email. RATs consisted of 6–7 boards-style multiple-choice questions from the American Academy of Pediatrics’ Pediatrics Review and Education Program. After the IRAT and GRAT were completed, questions and their answers were discussed and feedback was provided within the large group. Next, teams engaged in application exercises using clinical cases about each topic, which required trainees to make diagnostic and management decisions; for example, for the sports physicals session, teams reviewed actual clinical cases of adolescent children and had to decide how they would complete the sports physical form and whether they would allow the child to participate in high school level sports. Each team concurrently worked on the same clinical case through discussion at their tables. Then, the teams simultaneously presented their solutions to the large group and discussion ensued between teams to explain clinical reasoning and debate responses. At the conclusion, facilitators gave a brief verbal summary of the topics discussed during the session.

**Table 2:** Core design elements of team-based learning for pediatrics noon conferences

Core design element	Execution in resident noon conferences
Team Formation	• Teams formed based on clinical rotation
	• Tables assigned to guide team assembly
Readiness Assurance Tests (RAT)	• RAT designed based on published boards PREP questions (American Academy of Pediatrics’ Pediatrics Review and Education Program)
Immediate feedback	• Participants report answers to RAT and application exercise simultaneously
	• Immediately followed by large group discussion of correct answers and key principles, guided by facilitators
Sequencing of in-class problem solving	• Answers to the GRAT and Application Exercise were first discussed within teams, then discussed as a large group between all teams
Four Ss: *Significant problem* *Same problem* *Specific choice* *Simultaneous reporting*	• *Significant problem*: application exercise cases were drafted to be realistic, common diagnostic/management issues in pediatrics, RAT was composed of PREP questions
	• *Same problem*: all groups worked on the same problems
	• *Specific choice*: RAT- multiple choice questions, Application exercise- specific diagnostic/management questions about patient cases
	• *Simultaneous reporting*: RAT- groups “report out” by holding up large cut out letters simultaneously, Application exercise- groups write out their answer on white board
Incentive structure	• Competition between groups for points at each session
	• Cupcake prize for winning team after final session
Peer review	• Peer feedback was facilitated among and between groups during GRAT, application exercise and large group discussion

At the end of the three sessions, incentives in the form of food were given to the team who had the most points. Points were earned for the correct answers on the IRAT, GRAT, and application exercise, as judged by the chief residents. No grades were assigned for these sessions.

A pre-post design was used to evaluate feasibility, learner satisfaction, and knowledge acquisition. Before the first session, participants were surveyed about their experience with TBL, based on a three-point scale (none, some, several) (Additional file 1). After the last session, an anonymous questionnaire assessed residents’ reading completion rates (options: none, skimmed, half, entire article) as well as satisfaction and perceptions about engagement, knowledge acquisition, and desire for more TBL (five-point Likert scale for agreement). Likert scale data was analyzed by grouping responses into three categories: strongly agree / agree, neutral, and disagree / strongly disagree. Open-ended questions were utilized to assess strengths and areas for improvement (Additional file 2). Both the pre and post-assessment were developed by the authors. Attendance and IRAT/GRAT scores were recorded. Chief residents observed all sessions to assess strengths and challenges; after the final TBL session, the facilitators debriefed with the chief residents about the sessions and recorded notes. Quantitative analysis included descriptive statistics and Pearson Correlation. Qualitative analysis was conducted for open-ended questions utilizing an interactive process based on grounded theory principles [14, 15]. One author reviewed responses and developed codes independently. These codes were discussed and revised by the two authors until key themes were established and agreed upon. University of Chicago IRB deemed this study exempt.

## Results

There were 47 unique participants (36 residents and 11 medical students), with 29–33 learners per session. One-third of residents (36%, 13/36) attended all three TBL sessions and an additional 27% (10/36) attended two; the medical student rotation switched during this two-week block so each medical student attended only one of the TBL sessions. Twenty-nine participants completed the pre-questionnaire (62%, 29/47) and 27 (57%, 27/47) completed the post-questionnaire; the proportion of resident respondents was 83% (pre) and 59% (post), respectively.

### Feasibility

Most participants (55%, 16/29) were not familiar with TBL before this series. For preparation, 11% (3/27) of participants read the entire article for both sessions; more than one-third skimmed or did not read the article before the sessions.

Each TBL session lasted 45 min, rather than the planned one-hour, due to participant delays from obtaining food or clinical responsibilities as well as time required to organize the teams. The facilitators regularly attempted to maintain forward flow of the conference, however at times these efforts required shortening the time discussion to ensure all TBL components were included.

### Learner satisfaction

**Fig. 1:**
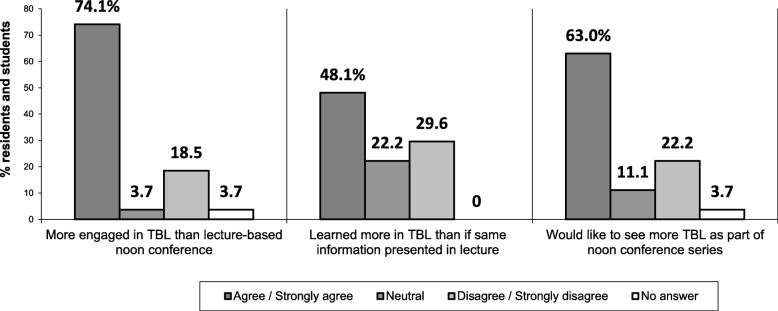
Learner perspectives of TBL compared to lecture-based noon conferences (*n* = 27)

Overall, 66.7% (18/27) of learners were satisfied with TBL sessions (see Fig. 1). Several learners appreciated the collaboration, teamwork, and critical thinking. One participant liked the “opportunity to work with residents at different levels and observe their approach to clinical scenarios.”

Learners desired additional time for each TBL component, describing that they “always felt rushed” and “needed more time to discuss as a team.” They proposed fewer activities/questions per session or longer sessions. Several participants suggested removing the IRAT, while others indicated it was a strength that provided the “opportunity to test our knowledge first, prior to attempting questions together.”

Learners actively participated in all TBL components. Most learners (74%, 20/27) reported more engagement during TBL than traditional conferences (see Fig. 1). One resident described TBL as “more interactive than typical noon conference” and another stated they “couldn’t tune out.” Chief residents and facilitators also observed higher levels of learner engagement than lecture-based conferences.

The majority (63%, 17/27) wanted more TBL, particularly those who felt more engaged and those who perceived they learned more in TBL versus traditional conferences (Pearson correlation, r = 0.914 and r = 0.771, *p* < 0.01).

### Knowledge acquisition

Mean IRAT and GRAT scores were: 57.1 (SD = 12.1) and 66.7 (SD = 17.3) for sports physicals and 45.2 (SD = 26.8) and 77.8 (SD = 22.8) for menstrual disorders, respectively. Nearly half of participants (48%, 13/27) perceived they learned more with TBL, as compared to lecture-based conferences.

## Discussion

Our study is one of the few to apply TBL in one-hour residency conferences. We demonstrate it leads to greater satisfaction and engagement among learners compared with traditional lectures, however substantial time constraints limit its feasibility during one-hour conferences. These results support findings of prior residency-based TBL studies [5, 11] and align with residents’ preferences for active learning [16].

It proved challenging to incorporate all TBL components in a one hour conference, in part because sessions were limited to 45 min in our real-world application. Due to the time limitations, discussion was truncated, potentially limiting positive outcomes. [1, 2] This challenge has not been previously described, because TBL has traditionally been utilized in 2–3-h blocks [5]. However, importantly, the general surgery curriculum sessions utilizing TBL were extended from 1 h to 1.75 h after the first year, suggesting similar time pressures [11]. The impact of time suggests that TBL may be better suited for longer conferences to ensure that learners attain the learning objectives. Alternatively, future studies can explore TBL adaptations to avoid curtailing discussion, such as limiting topic scope or completing IRAT before the conferences.

Despite learner engagement during sessions, pre-conference preparation rates were low, consistent with other residency-based TBL studies [7, 17]. Given busy residency schedules, learners may lack motivation or time to read, leading to poor compliance. Effective approaches to support knowledge acquisition pre-conference must be delineated [5]. Videos for individuals to review may help increase completion, or alternatively team-oriented pre-work can be considered as it may foster peer pressure [9]. Further, because grades carry less relevance in graduate medical education, relevant motivators at the resident level must be considered that can incentivize completion of the pre-conference preparation. Acceptability of preparation may also increase as participants gain familiarity with TBL [17].

Participants had mixed perceptions about whether they learned more in TBL versus lectures, suggesting tension between breadth and depth of content [16]. TBL emphasizes knowledge application, promoting depth at the expense of breadth, in contrast to lectures. However, depth may have been limited in our sessions due to the time constraints. Breadth and depth should be balanced in educational conferences. Future studies should examine if TBL designed for a traditional noon conference will perform similarly to the typical lectures presented within these conferences.

This curriculum was piloted in one residency program at an academic medical center, thus limiting generalizability. Facilitators had not previously led TBL but participated in faculty development, thus had experience similar to faculty who may adopt TBL for conferences. Because preparation was low among residents and students, the failure to develop accountability among learners to complete the preparatory work limits potential knowledge acquisition and overall impact of the TBL sessions; thus, consideration must be given to motivators to improve preparation. Finally, objective tools to assess knowledge and engagement were not utilized in this study and may have shown concordant or discordant results; future studies should incorporate such tools to further compare individuals’ perceptions about knowledge acquisition and engagement with objective findings.

## Conclusions

Our study shows TBL has the potential to foster more active learning, learner engagement, and knowledge acquisition than lectures during one-hour conferences; however, it is not feasible in its current design. Future work is needed to adapt TBL to better fit constraints of one-hour sessions, encourage pre-conference preparation, and evaluate the impact of TBL on knowledge retention and teamwork skills among residents.

## Additional files

Additional file 1: Appendix 1 - Pre-assessment for Team-Based Learning During Residency Noon Conference. (DOCX 17 kb)

Additional file 2: Appendix 2 - Post-Assessment for Team-Based Learning During Residency Noon Conference. (DOCX 20 kb)

## Data Availability

The data analyzed for this study are available from the corresponding author on reasonable request.

## Abbreviations

GRAT: Group readiness assurance test
IRAT: Individual readiness assurance test
RAT: Readiness assurance test
TBL: Team-based learning
