# Supplementary material for: A pilot study of team-based learning in one-hour pediatrics residency conferences
Source: BMC Med Educ. 2019 Jul 18;19:266. doi: 10.1186/s12909-019-1702-z (PMC6637552; doi:10.1186/s12909-019-1702-z)
Supplement: Supplementary file 1 — Appendix 1 - Pre-assessment for Team-Based Learning During Residency Noon Conference. (DOCX 17 kb) [file 12909_2019_1702_MOESM1_ESM.docx]

Appendix 2: Pre-Assessment for Team-Based Learning During Residency Noon Conference

*Team-Based Learning -* **Pre-Assessment**

**Please circle your level of training**

MS3 MS4 PGY1 PGY2 PGY3 PGY4 Other

**Consider your current (or most recent) clinical team.**

**Please check the box that represents your level of agreement with each statement.**

|  | **Strongly disagree** | **Disagree** | **Undecided** | **Agree** | **Strongly agree** |
| --- | --- | --- | --- | --- | --- |
| 1. The team members communicate well with one another. |  |  |  |  |  |
| 2. Constructive feedback is given by the team. |  |  |  |  |  |
| 3. Team members are familiar with each other’s job responsibilities. |  |  |  |  |  |
| 4. The team uses effective decision making processes and problem solving skills. |  |  |  |  |  |
| 5. The team monitors and progresses the plan of care. |  |  |  |  |  |
| 6. The team can change or improve the way it goes about working on its tasks. |  |  |  |  |  |
| 7. Team members trust each other. |  |  |  |  |  |
| 8. Morale on this team is high. |  |  |  |  |  |
| 9. Team members support each other. |  |  |  |  |  |
| 10. There are no feelings among team members that might pull this team apart. |  |  |  |  |  |
| 11. The team resolves conflicts soon after they occur. |  |  |  |  |  |
| 12. I feel free to express my opinions. |  |  |  |  |  |
| 13. I have an influence on team decisions. |  |  |  |  |  |
| 14. Team members can openly discuss their own problems and issues. |  |  |  |  |  |
| 15. Team members show consideration for needs and feelings of other team members. |  |  |  |  |  |
| 16. Team members receive recognition for individual performance. |  |  |  |  |  |

**Please circle the number that represents your experience with team-based learning.**

| **1. Rate your prior experience with participating in team-based learning (TBL):** | | | | |
| --- | --- | --- | --- | --- |
| 1 |  | 2 |  | 3 |
| *I have never participated as a learner in a TBL session* |  | *I have occasionally participated as a learner in a TBL session* |  | *I have frequently participated*  *as a learner in a TBL session*  *(either currently or in the past)* |
| **2. Rate your familiarity with the steps/phases of team-based learning (TBL):** | | | | |
| 1 |  | 2 |  | 3 |
| *No or minimal knowledge of TBL as an instructional method* |  | *Intermediate knowledge of TBL; I understand the basic process / phases of TBL* |  | *Expert knowledge of TBL as an instructional method; I can clearly explain each of the phases of TBL* |
