# Supplementary material for: A pilot study of team-based learning in one-hour pediatrics residency conferences
Source: BMC Med Educ. 2019 Jul 18;19:266. doi: 10.1186/s12909-019-1702-z (PMC6637552; doi:10.1186/s12909-019-1702-z)
Supplement: Supplementary file 2 — Appendix 2 - Post-Assessment for Team-Based Learning During Residency Noon Conference. (DOCX 20 kb) [file 12909_2019_1702_MOESM2_ESM.docx]

Appendix 2: Post-Assessment for Team-Based Learning During Residency Noon Conference

*Team-Based Learning -***Post-Assessment**

**Please circle your level of training.**

MS3 MS4 PGY1 PGY2 PGY3 PGY4 Other

**Please circle the statement that matches your prior experience with team-based learning.**

I had never participated I had previously participated I have previously participated

in a TBL session in 1-2 TBL sessions in several TBL sessions

***Please share your thoughts about the team-based learning sessions in the noon conference series.***

**Please indicate how much of the pre-work reading you completed prior to each session.**

|  | *Did not read article before session* | *Skimmed article* | *Read*  *½ article* | *Read entire article* | *Did not attend session* |
| --- | --- | --- | --- | --- | --- |
| Article: “Medical Conditions Affecting Sports Participation” |  |  |  |  |  |
| Article: “Menstrual Disorders” |  |  |  |  |  |

**Please rate your satisfaction overall and with each of the team-based learning sessions.**

|  | *Very dissatisfied* | *Somewhat dissatisfied* | *Neutral* | *Somewhat satisfied* | *Very satisfied* | *Did Not Attend* |
| --- | --- | --- | --- | --- | --- | --- |
| Overall Team-Based Learning |  |  |  |  |  |  |
| Session 1: Introduction / Survivor |  |  |  |  |  |  |
| Session 2: Sports Participation |  |  |  |  |  |  |
| Session 3: Menstrual Disorders |  |  |  |  |  |  |

**Please rate your level of agreement with each of the following statements.**

|  | *Strongly disagree* | *Disagree* | *Neutral* | *Agree* | *Strongly agree* |
| --- | --- | --- | --- | --- | --- |
| I was more engaged in the team-based learning sessions than in a typical noon conference |  |  |  |  |  |
| I learned more in the team-based learning sessions than if the same information was presented in a lecture in a typical noon conference |  |  |  |  |  |
| I would like to see more team-based learning sessions as part of the noon conference series |  |  |  |  |  |

**Please list two aspects you liked about the team-based learning sessions in the noon conference series.**

**Please suggest two ways to improve the team-based learning sessions in the noon conference series.**
